# Phylogeography of western Mediterranean *Cymbalaria* (Plantaginaceae) reveals two independent long-distance dispersals and entails new taxonomic circumscriptions

**DOI:** 10.1038/s41598-018-36412-1

**Published:** 2018-12-27

**Authors:** Pau Carnicero, Peter Schönswetter, Pere Fraga Arguimbau, Núria Garcia-Jacas, Llorenç Sáez, Mercè Galbany-Casals

**Affiliations:** 1 Systematics and Evolution of Vascular Plants (UAB) – Associated Unit to CSIC. Departament de Biologia Animal, Biologia Vegetal i Ecologia, Facultat de Biociències, Universitat Autònoma de Barcelona, 08193 Bellaterra, Spain; 2 Department of Botany, University of Innsbruck, Sternwartestrasse 15, 6020 Innsbruck, Austria; 3 Institut Menorquí d’Estudis, Camí des Castell 72, 07702 Maó, Spain; 4 Institut Botànic de Barcelona (IBB-CSIC-ICUB), Pg. del Migdia s/n, ES-08038 Barcelona, Spain; 5 Societat d’Història Natural de les Balears (SHNB), C/Margarida Xirgu 16, E-07011 Palma de Mallorca, Balearic Islands Spain

## Abstract

The Balearic Islands, Corsica and Sardinia (BCS) constitute biodiversity hotspots in the western Mediterranean Basin. Oligocene connections and long distance dispersal events have been suggested to cause presence of BCS shared endemic species. One of them is *Cymbalaria aequitriloba*, which, together with three additional species, constitute a polyploid clade endemic to BCS. Combining amplified fragment length polymorphism (AFLP) fingerprinting, plastid DNA sequences and morphometrics, we inferred the phylogeography of the group and evaluated the species’ current taxonomic circumscriptions. Based on morphometric and AFLP data we propose a new circumscription for *C*. *fragilis* to additionally comprise a group of populations with intermediate morphological characters previously included in *C*. *aequitriloba*. Consequently, we suggest to change the IUCN category of *C*. *fragilis* from critically endangered (CR) to near threatened (NT). Both morphology and AFLP data support the current taxonomy of the single island endemics *C*. *hepaticifolia* and *C*. *muelleri*. The four species had a common origin in Corsica-Sardinia, and two long-distance dispersal events to the Balearic Islands were inferred. Finally, plastid DNA data suggest that interspecific gene flow took place where two species co-occur.

## Introduction

Islands in the Mediterranean Basin harbour both high species diversity and endemism. For instance, from the around 5000 islands scattered in the Mediterranean Sea^1^ all the largest ones (i.e. Balearic Islands, Corsica, Crete, Cyprus, Sardinia and Sicily) constitute Mediterranean biodiversity hotspots^2^. Most of the Mediterranean islands are of continental origin and have been separated from the mainland and from each other through progressive geomorphological processes, often with posterior re-connections due to sea level variation^3^. Such processes are thought to have played an important role in the evolution and diversification of biota^4^.

The Balearic Islands, Corsica and Sardinia (hereafter termed BCS) originated from progressive splitting and rotation of the Hercynian belt, an Oligocene mountain range linking the Alps with the Iberian Peninsula that started to disintegrate around 30 Million years ago (Ma)^5^. The split between Corsica-Sardinia and the Balearic Islands occurred around 20 Ma^6^. Endemic plant species shared by Corsica, Sardinia and the eastern Balearic Islands (Mallorca and Menorca) have traditionally been considered relict palaeoendemic remnants of the Oligocene connections^3,7,8^. They were formerly named “Tyrrhenian endemisms” by Contandriopoulos and Cardona^7^, but we prefer to avoid this term given that the Tyrrhenian Sea is situated between Corsica, Sardinia, the Italian Peninsula and Sicily. To our knowledge, a date of origin consistent with the paleoendemics hypothesis has only been confirmed for *Helicodiceros muscivorus* (L.f.) Engler^9^. Bobo-Pinilla *et al*.^10^ have recently proposed a relict origin for the BCS endemic *Arenaria balearica* L., but the lack of absolute dating estimates did not allow for conclusive results. In most other dated phylogenies of plants showing this distribution pattern (e.g. *Arum pictum* L.f.^9^; *Thymus herba-barona* Loisel.^11^; *Cymbalaria aequitriloba* (Viv.) A.Chev.^12^, or other similar disjunctions in the western Mediterranean Basin^13,14^, palaeoendemism hypotheses had to be rejected (but see Magri *et al*.^15^. Instead, long-distance dispersal (LDD) was invoked as a probable explanation for the present distribution pattern, since no land connections have been reported between the Balearic Islands on the one hand and Corsica and Sardinia on the other hand after their split.

Pleistocene climatic oscillations caused massive changes of the sea level that led to repeated appearance of land bridges between some islands during cold stages, and to transgressions reducing the area of islands during interglacials^3,16^. During glaciation periods Corsica-Sardinia and the eastern Balearic Islands (Mallorca, Menorca and Cabrera), respectively, formed single landmasses. The resultant connections have often been used to explain phylogeographic patterns and shared endemisms within each archipelago (e.g. Mansion *et al*.^17^; Salvi *et al*.^18^; Mayol *et al*.^19^).

**Figure 1:**
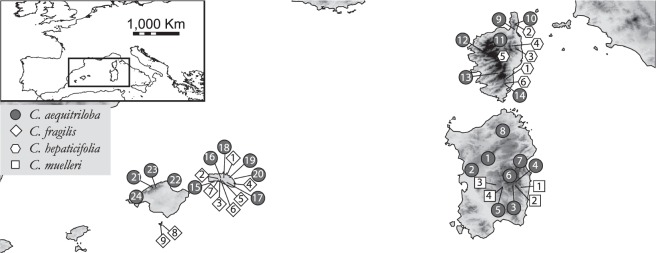
Sampling sites and population numbers. Taxonomy follows the taxonomic concept adopted in the present article, population numbers in the main text are preceded by a letter according to taxonomy (a: *C*. *aequitriloba*, f: *C*. *fragilis*, h: *C*. *hepaticifolia*, m: *C*. *muelleri*, see Supplementary Table S1). Map generated from the altitude layer obtained from http://www.worldclim.org ^132^ and modified using QGIS v2.18.2^133^.

One of the genera with highest number of endemics in BCS is *Cymbalaria* Hill (Plantaginaceae), a Mediterranean genus comprising ca. 17 taxa, four of which are endemic to BCS^20^ (Fig. 1). The BCS endemics form an independent lineage of presumably polyploid origin that diversified during the Pliocene and the Pleistocene, as revealed by a nuclear ribosomal DNA (nrDNA) phylogeny^12^. However, interspecific phylogenetic relationships were poorly resolved, and therefore using more variable molecular markers such as AFLPs would be desirable. Chromosome counts suggest that the BCS endemics are hexa- to octoploids (2n = 42, 56), whereas all other *Cymbalaria* species are diploids or tetraploids (2n = 14, 28^20^ and references therein). While *C*. *aequitriloba* is widely distributed throughout BCS and occurs in all main islands except for the western Balearic Islands Eibissa and Formentera, the other three species are single island endemics according to the current taxonomic treatments^20,21^. This lineage is therefore an excellent example to understand the mechanisms underlying the endemism patterns in BCS.

The two single island endemics from Corsica and Sardinia have been traditionally considered as clearly distinct species. *Cymbalaria hepaticifolia* Wettst. (2n = 56^20^) occurs in the mountains of Corsica, from subalpine habitats to fresh and humid forests^22,23^. *Cymbalaria muelleri* (Moris) A.Chev., comprising ca. ten known populations, is a hexaploid (2n = 42^24^) endemic of the mountains of central Sardinia^25^.

*Cymbalaria fragilis* (J.J.Rodr.) A.Chev. is a critically endangered (CR) octoploid (2n = 56^26^) species endemic to Menorca^27^. It has been treated either as a subspecies of *C*. *aequitriloba*^20,28^ or as a separate species^21^. Furthermore, the circumscription of this taxon is uncertain. According to Güemes^21^, *C*. *fragilis* is characterized by several distinctive features such as white to pale violet flowers, fleshy leaves and fragile stems, dense hairiness and finely alveolate to nearly smooth seeds. However, some populations in Menorca, formerly identified as *C*. *fragilis*^29^, and Cabrera exhibit all distinctive features but have deeply alveolate seeds similar to those of *C*. *aequitriloba*^21^. Seed ornamentation has been regarded as highly relevant in the taxonomy of the tribe Antirrhineae^20,30–32^ and this character was later considered sufficient by Güemes^21^ to attribute these populations to *C*. *aequitriloba*. The latter is the most widespread species among the four BCS endemics, occurring in the eastern Balearic Islands, Corsica, Sardinia and the Tuscan Archipelago. Octoploid counts (2n = 56) have been reported from Corsica and the Balearic Islands^33–35^; older hexaploid counts (2n = 42^20^) were considered erroneous by Verlaque *et al*.^35^. Indeed, the reference cited by Sutton^20^ for the hexaploid counts^36^ does not report any chromosome number for *C*. *aequitriloba*, and we were unable to trace any other hexaploid count in the literature. *Cymbalaria aequitriloba* exhibits high morphological variability, which is the reason why sparsely hairy forms from Mallorca have sometimes been confused with *C*. *hepaticifolia*^37^. Some morphological extremes were described as separate taxa, but they were later synonymised with *C*. *aequitriloba*^20^. Nuclear ribosomal and plastid DNA sequences, however, did not support the species’ monophyly^12^, and it is thus doubtful whether *C*. *aequitriloba* is indeed a single taxon. Some authors, based on chromosome number information, considered *C*. *aequitriloba* an example of a palaeoendemism^3,7,8^, although some of its aforementioned features, such as its high morphological variability, do not fit with the classical definition of the term^38^. Most importantly, its recent origin inferred from a dated phylogeny rejected that hypothesis^12^.

Here, we focus on the phylogeography and systematics of the BCS endemics of the genus *Cymbalaria*. We use a combination of AFLPs, plastid DNA sequences and morphometrics to tackle the following questions: (1) Are genetic and morphological data congruent and do they support the current taxonomy? (2) Specifically, is *C*. *aequitriloba* monophyletic based on AFLP data? (3) What is the appropriate taxonomic rank and circumscription of *C*. *fragilis*? (4) What was the pattern of colonization of the BCS endemics among Sardinia, Corsica and the Balearic Islands?

## Results

### AFLPs

In total, 834 AFLP fragments were scored for 304 individuals (Supplementary Table S1), of which 779 polymorphic, high-quality, reproducible AFLP-fingerprints were obtained. Fifty-five fragments with singular presences or absences were excluded. The initial error rate^39^ before the exclusion of non-reproducible fragments was 3.17%. The final dataset for *C*. *aequitriloba* comprised 175 individuals and 557 AFLP fragments.

**Figure 2:**
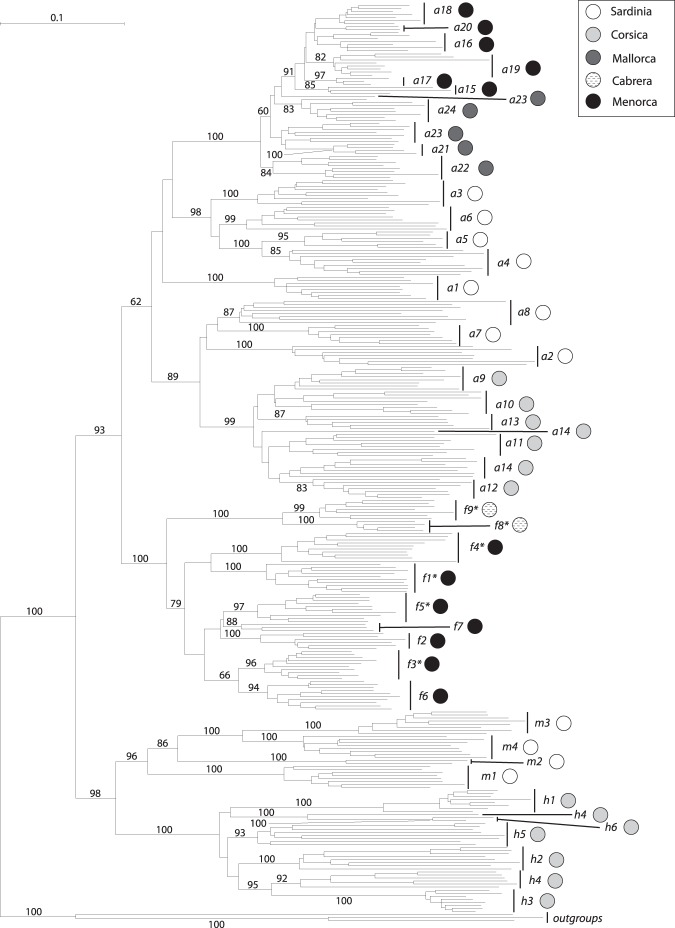
AFLP analyses of *C*. *aequitriloba*, *C*. *fragilis*, *C*. *hepaticifolia* and *C*. *muelleri*, Neighbour-Joining tree. Numbers above branches are bootstrap support values (>60%) derived from a Neighbour-Joining analysis. Asterisks indicate *C*. *fragilis* populations previously considered *C*. *aequitriloba*. Populations are numbered as in Fig. 1 and Table S1. Circles next to population names indicate the island of origin.

The NJ analysis revealed that the four taxa analysed constituted a monophyletic group with high bootstrap support (BS 100%, Fig. 2). The main split in the NJ tree separated *C*. *aequitriloba* and *C*. *fragilis* from *C*. *hepaticifolia* and *C*. *muelleri*. Populations of *C*. *aequitriloba* formed a clade (BS 62%) excluding all populations with distinctive features of *C*. *fragilis*, but bearing alveolate seeds (f1, f3, f4, f5, f8 and f9), which instead grouped with *C*. *fragilis* (BS 100%). One well supported clade each grouped populations of *C*. *hepaticifolia* (BS 100%) and *C*. *muelleri* (BS 96%). Individuals from the same population – with the exception of populations a14, a23 and h4 – formed monophyletic groups. The *C*. *fragilis* clade showed a main split between the populations from Menorca (f1 to f7, BS 79%) and those from Cabrera traditionally included in *C*. *aequitriloba* (f8 and f9, BS 100%).

The most frequent species tree topology (61%) obtained by the SNAPP analysis coincided with the NJ analysis (Supplementary Fig. S1). However, a second most frequent topology (30%) indicates different relationships among the species: *C*. *hepaticifolia* diverged first, followed by the differentiation of *C*. *muelleri*, which is sister to the *C*. *aequitriloba*-*C*. *fragilis* clade. Both topologies showed how the diversification of the four western *Cymbalaria* species occurred roughly simultaneously. Three alternative topologies represented less than 10% of the total number of trees.

**Figure 3:**
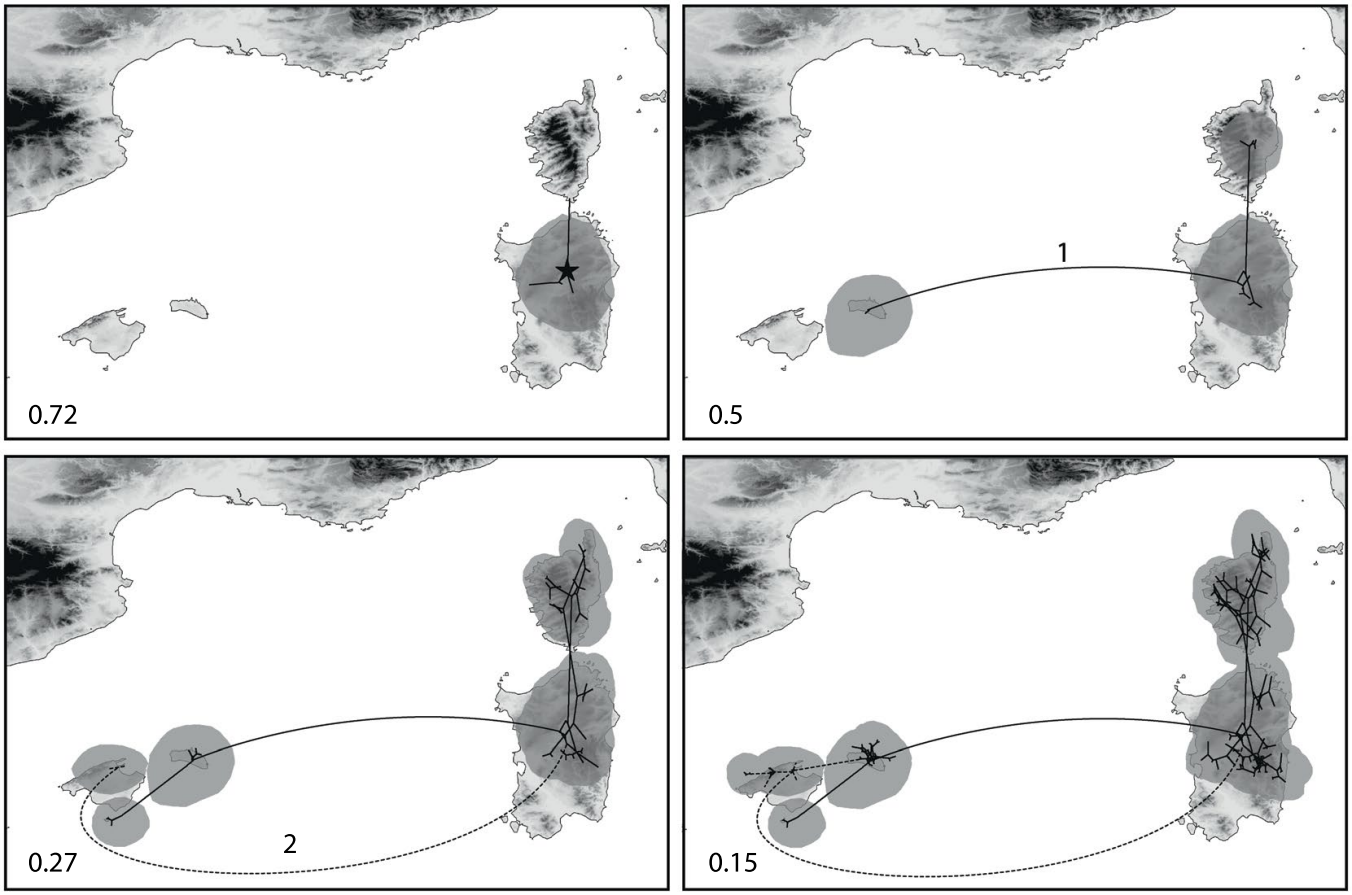
Snapshots of estimated ancestral node areas in the Maximum Clade Credibility tree obtained from the BEAST continuous phylogeographical analysis of AFLP data at different time horizons as visualized using the software SPREAD. The 80% highest posterior density areas for nodes are indicated as grey polygons. The star in the upper left panel indicates the starting point of diversification and the relative time scale of diversification is indicated by numbers (1 = origin, 0 = present). Numbers indicate LDD events to the Balearic Islands leading to (1) the origin of *C*. *fragilis* and (2) a range expansion of *C*. *aequitriloba*. Lines indicating the two LDD events were modified to prevent them from crossing. Maps generated from the altitude layer obtained from http://www.worldclim.org^132^, and modified using QGIS v2.18.2^133^.

The results obtained by the Bayesian analyses with BEAST were congruent with the NJ analysis, minor differences concerned nodes with low support (Supplementary Fig. S2). The origin of diversification was estimated to be in northern Sardinia (Fig. 3), from where dispersal events to southern Sardinia, Corsica and the Balearic Islands occurred. Later, further dispersals from Sardinia to Corsica and the Balearic Islands (Mallorca) took place.

A hierarchical AMOVA (Supplementary Table S2) assigned 28.7% of the entire variation to the among-taxa component. In *C*. *aequitriloba* the highest variation among populations within an island was found in Sardinia (44.7%), and the lowest in Corsica (27.7%). Gene diversity expressed as π_n_ (Supplementary Table S3) varied from 0.028 in population a17 of *C*. *aequitriloba* from Menorca to 0.164 in population h5 of *C*. *hepaticifolia* from Corsica. No differences were found between taxa. For *C*. *aequitriloba*, π_n_ was significantly lower in the Balearic Islands than in Corsica and Sardinia.

**Figure 4:**
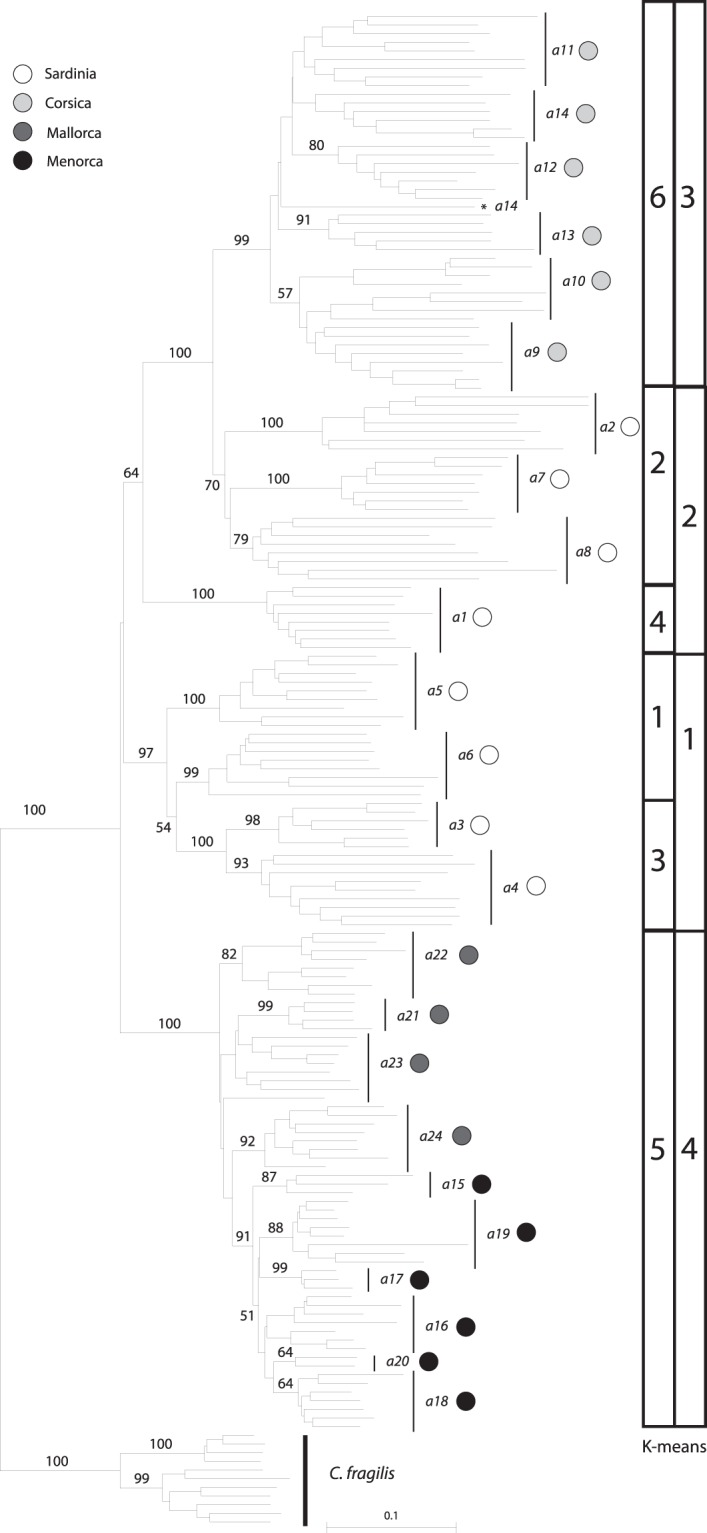
Neighbor-Joining tree based on AFLP data generated for *C*. *aequitriloba* following the taxonomic concept adopted in the present study. Distributions of clusters identified by nonhierarchical K-means clustering at K = 4 and K = 6 are shown to the right. Circles next to population names indicate the island of origin.

In the separate NJ analysis for *C*. *aequitriloba* (BS 100%, Fig. 4) the populations from the Balearic Islands formed a clade (BS 100%), which contained a subclade comprising populations from Menorca (a15–a20, BS 91%). Populations from Corsica (a9–a14) formed a supported clade (BS 99%) whereas populations from Sardinia were distributed in three main clades (a1, BS 100%; a2, a7 and a8, BS 70%; a3–a6, BS 97%). Nonhierarchical K-means clustering resulted in an optimal separation of the dataset into four groups (Fig. 4) composed by 1) the Sardinian populations a3–a6; 2) the Sardinian populations a1, a2, a7 and a8; 3) all Corsican populations; and 4) all Balearic populations. At K = 6 populations a1–a8 from Sardinia were split into four clusters, whereas the other two groups included all Corsican and all Balearic populations, respectively (Fig. 4).

### Plastid DNA Sequences

**Figure 5:**
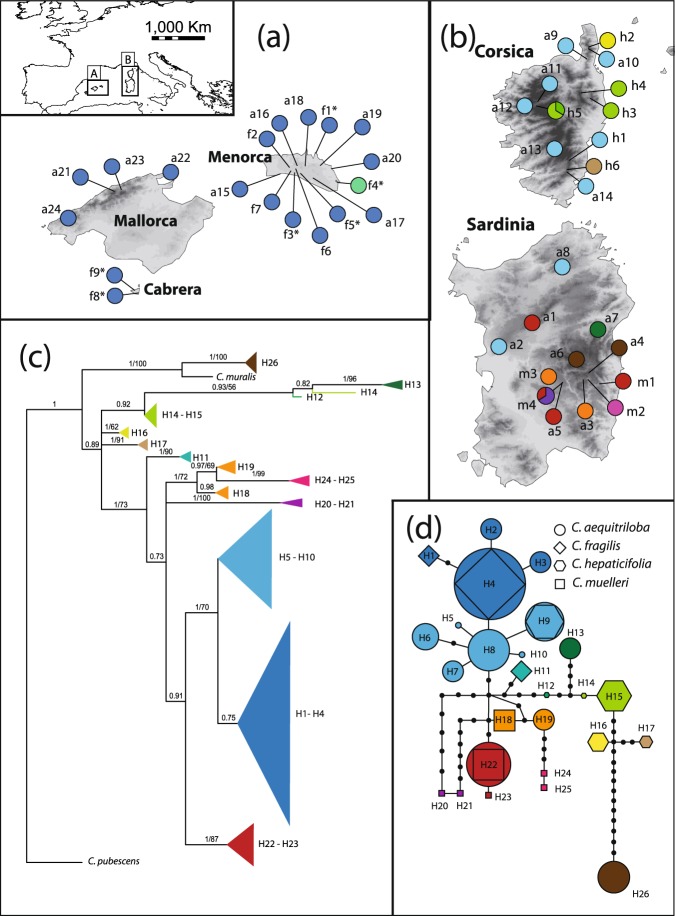
Sampled populations and patterns of plastid DNA (*ndhF* and *rpl32-trnL(UAG)*) variation. Colours correspond to groups of closely related haplotypes. Taxonomy follows the taxonomic concept adopted in the present article (a: *C*. *aequitriloba*, f: *C*. *fragilis*, h: *C*. *hepaticifolia*, m: *C*. *muelleri*). Maps generated from the altitude layer obtained from http://www.worldclim.org132, and modified using QGIS v2.18.2^133^. (**a**,**b**) Pie charts indicate the proportions of haplotype groups sampled in each population. Asterisks indicate populations exhibiting mostly distinctive features of *C*. *fragilis* but previously assigned to *C*. *aequitriloba* owing to the presence of alveolate seeds. (**c**) Phylogram from the Bayesian Inference analysis. Numbers above branches indicate Bayesian posterior probabilities/Maximum parsimony bootstrap support values. Haplotypes are given for each lineage next to the tips, numbering corresponds to Table S1. (**d**) Statistical parsimony haplotype network. Black dots indicate hypothetical unsampled haplotypes, numbering corresponds to Table S1.

The concatenated *ndhF* and *rpl3*2*-trnL(UAG)* sequences consisted of 2776 aligned positions. We found 26 plastid haplotypes that differed by one to 14 substitutions including codified indels (Fig. 5). There was no clear correlation with taxonomy; instead, some geographically close populations of different taxa shared haplotypes. For instance, haplotype 4 was found in almost all populations of *C*. *aequitriloba* and *C*. *fragilis* from the Balearic Islands, haplotype 9 was found in two populations of *C*. *aequitriloba* and one population of *C*. *hepaticifolia* from Corsica, and haplotype 22 was found in two populations of *C*. *aequitriloba* and one population of *C*. *muelleri* from southern Sardinia (Fig. 5). The Balearic Islands clearly showed low haplotype diversity in comparison to Corsica and Sardinia. Relationships inferred by MP and Bayesian analyses were congruent and consequently we only show the topology from the Bayesian analysis (Fig. 5). Two main clades were found in the tree: the first contains the strongly divergent haplotype 26 from Sardinia and a haplotype of *C*. *muralis* (PP 1; BS 100%), and it is sister to all other haplotypes found in BCS (PP 0.89). Phylogenetic relationships among haplotype groups (colour-coded in Fig. 5) were in general statistically weakly supported, whereas relationships among closely related haplotypes reflected the pattern of the parsimony network.

### Morphometric analyses

Correlation coefficients did not exceed 0.95 for any pair of characters; therefore, all characters were retained for further analyses (Supplementary Table S4).

#### Dataset 1

**Figure 6:**
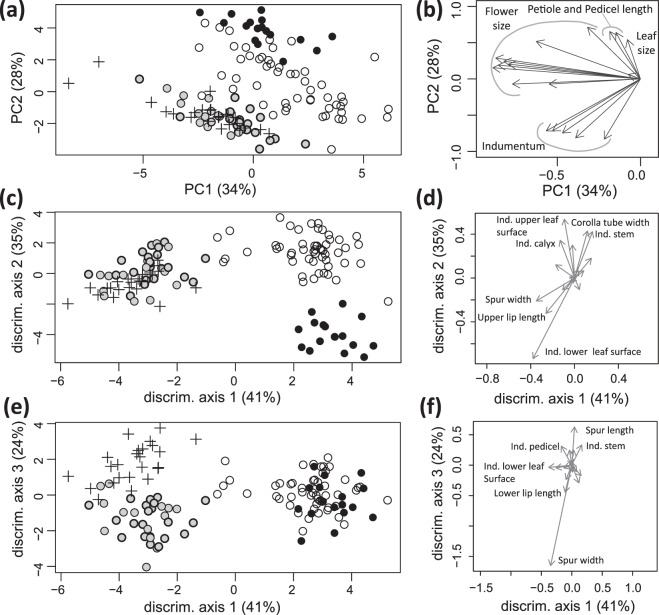
Ordination diagrams of morphometric analyses of 18 floral and vegetative characters obtained for 129 specimens (dataset 1) of *C*. *aequitriloba* (empty circles), *C*. *fragilis* (grey dots), *C*. *hepaticifolia* (black dots) and *C*. *muelleri* (crosses). Grey dots with thicker outline indicate *C*. *fragilis* specimens previously considered *C*. *aequitriloba*. (**a**,**b**) Principal component analysis. (**a**) Scatter plot of principal component scores for the first two components of morphological variation. (**b**) Character relationships and contribution to the first two axes. (**c**–**f**) Canonical discriminant analysis. (**c**,**e**) Scatter plots of discriminant scores for two combinations of the three discriminant axes. (**d**,**f**) Character relationships and contribution in the same ordination space as (**c**,**e**). Characters with high contributions in each combination are identified. (ind. = indumentum).

In the PCA, the first axis accounted for 34% of the variation and the second axis for 28%. The ordination diagram (Fig. 6a) suggested three clusters, two corresponded to *C*. *aequitriloba* and *C*. *hepaticifolia*, respectively, and a third one grouped *C*. *fragilis* and *C*. *muelleri*, including the controversial *C*. *aequitriloba* populations f1, f3, f4, f5, f8 and f9 from Cabrera and Menorca. The characters with most weight in the separation of the three groups were those related with indumentum density. The CDA (Fig. 6b–e) supported morphological differentiation of the four main AFLPs clades. The first two axes (41% and 35% of the explained variation) supported the three groups observed in the PCA, with some overlap between *C*. *aequitriloba* and *C*. *fragilis* plus *C*. *muelleri*. The characters with the highest contribution to these axes were derived from the leaf indumentum. The third axis (24%) supported the differentiation between *C*. *fragilis* and *C*. *muelleri*. The characters with the highest contribution to the third axis were spur length and width.

#### Dataset 2

The first PCA axis accounted for 44% of the variation and the second axis for 14%. The ordination diagram showed a structure similar to that retrieved by the analysis of dataset 1, albeit with stronger overlap of *C*. *aequitriloba* and the *C*. *fragilis* – *C*. *muelleri* group (Supplementary Fig. S3).

## Discussion

The combination of molecular and morphological data has been widely used to reevaluate systematics at different taxonomic levels (e.g.^32,40–42^). Specifically, many studies have used AFLP data and morphometrics to address taxonomic conflicts in groups of closely related plant taxa (e.g.^43–47^). In the case of *Cymbalaria*, AFLP (Fig. 2) and morphometric analyses (Fig. 6) allow for the recognition of four distinct entities within a previously identified monophyletic group of *Cymbalaria* species endemic to the Balearic Islands, Corsica and Sardinia^12^. These entities coincide with the current taxonomic circumscription of *C*. *hepaticifolia* and *C*. *muelleri*, but require novel circumscriptions of *C*. *aequitriloba* and *C*. *fragilis* (Figs 2 and 6).

### A novel circumscription of *C*. *fragilis* and *C*. *aequitriloba*

Güemes^21^ suggested that *C*. *fragilis* is restricted to three populations in southwestern Menorca, and assigned reports from the North and East of the island (populations f1 and f4) as well as those from Cabrera (populations f8 and f9) to *C*. *aequitriloba*, based on their alveolate seeds. However, these populations exhibit all distinctive morphological features of *C*. *fragilis* such as (1) villous indumentum throughout the vegetative parts, (2) highly fragile stems, petioles and pedicels, (3) fleshy leaves, (4) white to pale violet corollas, (5) spur proportionally shorter and wider than in *C*. *aequitriloba* (ratio spur length/spur width 0.9–2.8), (6) seeds, which are often smaller than those of *C*. *aequitriloba* (0.6–0.9 mm long, vs. 0.8–1.2 mm long in *C*. *aequitriloba*, see identification key), as well as (7) a late flowering time compared to *C*. *aequitriloba* (June as compared to early spring in *C*. *aequitriloba* from the same altitude). Interestingly, in population f6 we observed both types of seed ornamentation. In the AFLP (Figs 2 and 4) as well as in the morphometric analyses (Fig. 6), these populations clustered with *C*. *fragilis*. Therefore, *C*. *fragilis* must be redefined to include populations with the before-mentioned distinctive morphological characters from Menorca and Cabrera in spite of their aberrant seeds. Seed ornamentation – in this particular case – must thus be considered a variable character without taxonomic value. The strongly supported monophyly in the AFLP analyses (Fig. 2) together with the clear morphological differentiation (Fig. 6) and the overlapping distributions of *C*. *fragilis* and *C*. *aequitriloba* in Menorca do not match the definition of subspecies^48–50^. In contrast, the available evidence strongly suggests treating *C*. *fragilis* as a separate species in accordance with the original description^51^, rather than as a subspecies of *C*. *aequitriloba*^20,52^. The proposed change of circumscription brings about a change in the species’ threat category according to IUCN^53^ criteria. We sampled all known populations of *C*. *fragilis* to date, updating the number of populations from three to nine, and therefore suggest that Near Threatened (NT) would be a more adequate category than the formerly applied Critically Endangered (CR)^27^.

### A Corso-Sardinian common ancestor for the BCS endemics

Spatial reconstruction of evolutionary dynamics using relaxed random walks (Fig. 3) suggests a Sardinian origin of the BCS endemic species of *Cymbalaria*. The low haplotype diversity found in the Balearic Islands (Fig. 5) is congruent with a Sardinian origin, but does not allow to reject the alternative hypothesis of a Corsican origin. An east (Corsica-Sardinia) to west (Balearic Islands) pattern of colonization has been also suggested for the BCS endemics *Arum pictum*^9^ and *Thymus herba-barona*^11^.

AFLP and morphological data confirmed *C*. *hepaticifolia* and *C*. *muelleri* as two well differentiated species (Figs 2 and 6). They diverged from a common ancestor probably via an allopatric speciation event, as suggested by their current distribution areas (Fig. 1, Supplementary Fig. S1). However, the SNAPP analysis revealed an alternative topology supported by 30% of trees, which resolved *C*. *hepaticifolia* as sister of the other BCS endemics, and *C*. *muelleri* as sister of *C*. *fragilis* and *C*. *aequitriloba*. This uncertainty could be caused by rapid simultaneous diversification of the BCS endemics, as suggested for the initial diversification of *Cymbalaria*^12^. Indeed, rapid diversification rates after colonisation of new areas by polyploid lineages have been previously suggested^54,55^. Alternatively, the closer relationship of *C*. *muelleri* with the *C*. *aequitriloba-C*. *fragilis* clade could have been caused by gene flow between *C*. *muelleri* and *C*. *aequitriloba* or its ancestor in Sardinia. Plastid sequences revealed, indeed, close relationships among the haplotypes found in *C*. *hepaticifolia* and *C*. *muelleri* and those of sympatric *C*. *aequitriloba* populations (Fig. 5).

Both the strongly reduced haplotype diversity of *C*. *fragilis* in comparison with Corsican and Sardinian populations of the three other studied species (Fig. 5) and the continuous phylogeographic analysis (Fig. 3) support the hypothesis that a LDD event to the Balearic Islands led to the origin of *C*. *fragilis*. Genetic diversity of *C*. *fragilis* was not significantly lower than that of *C*. *aequitriloba* (Supplementary Table S3), suggesting that the LDD event was ancient enough to allow for a recovery of genetic diversity^56^. *Cymbalaria aequitriloba* has its centre of genetic diversity in Sardinia, as shown by the highest among-population variation in the AMOVAs (Supplementary Table S2), the significantly higher genetic diversity observed in Sardinian populations as compared to populations from the other islands (Supplementary Table S3), the high haplotype diversity (Fig. 5) and the high number of separable AFLP gene pools (Fig. 4).

### Phylogeography of *C. aequitriloba* and *C. fragilis*

*Cymbalaria aequitriloba* exhibits a higher genetic diversity in Sardinia than in Corsica, as shown by the higher number of plastid haplotypes and AFLP clusters (Figs 4 and 5). This is most probably explained by Pleistocene glaciation periods, which had a stronger effect in Corsica than in Sardinia as illustrated by the large glaciers documented in Corsica during the Last Glacial Maximum^57^ (18,000 years ago), and could have caused local extinctions^58,59^. Later range expansion might have occurred from either lowland Corsican or Sardinian refuge areas, via land bridges connecting both islands multiple times during the Pleistocene^60,61^ or across the narrow Bonifacio strait separating Corsica and Sardinia^62^. Populations from northern and western Sardinia showed a high genetic similarity with Corsican populations (Figs 4 and 5), supporting contacts between the populations of the two islands as shown for *Cistus creticus* L.^62^.

*Cymbalaria* reached the Balearic Islands via two independent LDD events, as supported by the NJ tree (Fig. 2) and the continuous phylogeographic analysis (Fig. 3), combined with evidence of a relatively young origin of *Cymbalaria* BCS endemics, *ca*. 15–20 myr later than the last land connection between Corsica-Sardinia and the Balearic Islands^12^. The first LDD events ultimately led to the origin of *C*. *fragilis* and the second caused a range expansion of *C*. *aequitriloba*. These two events were also supported by the shorter terminal splits depicted in the NJ tree for all Balearic populations indicating strong similarity among individuals (Figs 2 and 4). Significantly lower average genetic diversity of Balearic populations of *C*. *aequitriloba* as compared to those from Corsica and Sardinia also support the hypothesis of a recent LDD event (Supplementary Table S4). In addition, the strongly reduced haplotype diversity in the Balearic Islands (Fig. 5) may evidence a strong bottleneck resulting from a founder event (e.g., Schönswetter *et al*.^63^, Burnier *et al*.^64^). With the exception of haplotype H11, the close phylogenetic relationships among haplotypes from the Balearic Islands (Fig. 5c) suggest a common ancestral haplotype for *C*. *aequitriloba* and *C*. *fragilis*. This common ancestor might have occurred in western and northern Sardinia as shown by the BEAST continuous phylogeographic analysis and the close phylogenetic relationship between haplotypes H1–H4 from the Balearic Islands and H5–H10 from Corsica and Sardinia (Figs 3 and 5). In the same line, the geographic setting supports a dispersal from western Sardinia, for instance from the surroundings of population a2. It is the closest to the Balearic Islands and thrives on the summit of the Monte Ferru massif, at 1050 m altitude only 12 km from the coast. Alternatively, or in addition, at a later stage hybridization with *C*. *fragilis* could have led to the sharing of haplotype H4 across species boundaries^65^.

The history of the Balearic populations of both species was likely influenced by sea level variation during the Pleistocene. Land connections during glaciation periods^16^ might have aided *C*. *aequitriloba* and *C*. *fragilis* to disperse among the Eastern Balearic Islands until both species achieved their current distributions. However, during early Pleistocene interglacials (1.6–0.7 Ma) the sea rose for up to 100 m above current level, leading to considerable reduction of the surface of Menorca, division of Mallorca into two islands and transgression of most of Cabrera^19,66^. This was likely a major obstacle for the expansion of *C*. *fragilis* to Cabrera, since only a small area of that island protruded from the sea, suggesting that the colonization most probably occurred after this period. In any case, presence of well-supported clades in Menorca and Cabrera (Fig. 2) supports the current isolation.

In contrast to other members of the tribe Antirrhineae exhibiting winged seeds or adaptation to marine dispersal (e.g. *Linaria* Mill., *Cymbalaria longipes* (Boiss. & Heldr.) A.Chev.)^20^ neither *C*. *aequitriloba* nor *C*. *fragilis* show any apparent adaptation for LDD. Moreover, both species are at least partly achorous and actively prevent seed dispersal by positioning mature capsules in rock crevices^20^. Notably, very similar features were used as an argument against LDD for the BCS endemic *Arenaria balearica*^10^. However, the importance of non-standard vectors has been recognised^67,68^ and rare stochastic events such as LDDs have been highlighted as important drivers of organisms’ histories^69,70^. In fact, LDD has been invoked in several cases in spite of the apparent lack of particular adaptations (e.g. Guzmán and Vargas^71^; Dixon *et al*.^72^, Escudero *et al*.^73^, Santos-Gally *et al*.^74^, Piñeiro *et al*.^75^). In the case of *Cymbalaria*, the small size of seeds could have favoured LDD as a consequence of rare stochastic events, as have been invoked for *Androsace*^67^ or suggested as an *a priori* possibility for *A*. *balearica*^10^. The success in colonization after a LDD is thought to be governed by the availability of adequate niches rather than by dispersal abilities^4^. In addition, polyploids are better buffered against genetic bottlenecks and subsequent inbreeding resulting from founder effects^76^, a feature that could have helped *C*. *aequitriloba* and *C*. *fragilis* to successfully establish in the Balearic Islands.

Finally, our previous results^12^ strongly suggested that *C*. *aequitriloba* is not a palaeoendemic that originated and expanded its range when Corsica-Sardinia and the Balearic Islands were connected^6^ (~20 Ma), but rather colonized the Balearic Islands much later, 1–4 Ma. The results presented here support this statement and furthermore show that *C*. *aequitriloba* does not meet the criteria attributed to relict taxa, i.e., low levels of infraspecific morphological and genetic variation as a result of a long process of adaptation in refugial isolation, and taxonomic isolation due to extinction of close relatives^8,37^. In contrast, *C*. *aequitriloba* is highly morphologically variable^20,21^ (Fig. 6), genetically equally diverse as its closest relatives (Supplementary Table S3) and not taxonomically isolated. Obviously, knowledge of the temporal framework is crucial for any study of palaeoendemism, since it allows for evaluating the congruence between a biogeographic hypothesis and, for instance, the geomorphological distribution of landmasses. In at least two cases focussing on BCS, dating analyses have rejected the hypothesis of a continuous palaeodistribution in the Oligocene landmass followed by fragmentation (*A*. *pictum*^8^, *T*. *herba-barona*^11^). To date, molecular dating has resulted in a temporal framework congruent with a continuous palaeodistribution only in the case of *H*. *muscivorus*^8^. Although Bobo-Pinilla *et al*.^10^ suggested *A*. *balearica* to be a palaeoendemic, the lack of absolute time estimates prevented them from firmly rejecting a more recent origin. Often, dating analyses are revealing more recent divergent times than formerly thought (e.g. Salvo *et al*.^13^, Fernández-Mazuecos *et al*.^14^) and are thus increasingly shifting the biogeographic paradigm for the Mediterranean area.

### Incongruence between AFLP and sequence data

The monophyly of each of the four species in the AFLP analyses (Fig. 2) suggests that reproductive isolation is effective at present. However, interspecific gene flow appears possible due to the close proximity between populations of *C*. *aequitriloba* and *C*. *hepaticifolia* in Corsica, and between *C*. *aequitriloba* and *C*. *muelleri* in Sardinia (P.C. & M.G., personal field observations). A few incongruities between mostly nuclear-derived AFLPs^77^ and maternally inherited plastid DNA sequences^78^, could indicate past reticulation events. Although incomplete lineage sorting (ILS) of ancient polymorphisms cannot be ruled out as cause for these incongruities^79–83^ hybridization appears more likely due to the grouping of haplotypes retrieved from geographically close populations of different taxa^84,85^. The most obvious case is the occurrence of haplotype 26 otherwise characteristic of the phylogenetically distant (cf. Carnicero *et al*.^12^) *C*. *muralis* in populations a4 and a6 of *C*. *aequitriloba* from Sardinia (Fig. 4). *Cymbalaria muralis*, a diploid species native to the Apennine and northern Balkan Peninsulas, is naturalized almost worldwide in temperate areas^20^. The population of *C*. *muralis* used here is from the same area in Sardinia where haplotype 26 was found; hybridization resulting in chloroplast capture of a *C*. *muralis* haplotype is, therefore, likely.

Two statistically supported incongruities were found between the AFLP dataset and the previous phylogeny^12^. The latter retrieved a clade formed by *C*. *aequitriloba* accessions from Corsica and *C*. *hepaticifolia*, and inferred a common ancestor for *C*. *fragilis* and *C*. *muelleri*; these two clades did not appear in the AFLP analysis (Fig. 2). Although nrDNA and AFLPs have been shown to yield mostly congruent signals^86^, incongruities have also been reported^87,88^. Here, we suggest that hybridization followed by concerted evolution of nrDNA towards one parental copy^79,89^, ILS and/or too little variation in nrDNA sequences are the most probable causes for the incongruities. As above argued for the plastid DNA data, we consider hybridization the most probable cause for the grouping of *C*. *aequitriloba* and *C*. *hepaticifolia* in the nrDNA tree, since they occur in the same areas and exhibit no obvious reproductive barriers (P.C. & M.G., personal field observations). In contrast, ILS or insufficient variation among nrDNA sequences seem the most probable causes for incongruence affecting *C*. *muelleri* and *C*. *fragilis*, since they have non-overlapping distribution areas. In the same line, low variability of DNA sequences has previously been reported as cause for incongruence between DNA sequences and AFLP fingerprints^90^. Therefore, we consider the aforementioned relationships retrieved from the nrDNA phylogeny to be spurious. Similarly, it has been stated that AFLPs explain better phylogenetic relationships among closely related species, especially when dealing with complex evolutionary processes such as polyploidy and hybridization^86^.

### Identification Key

Here we provide a dichotomous key to all *Cymbalaria* species occurring in BCS.

1. Leaves and stems glabrous or only with occasional hairs on young organs……….……….……….……… **2**
  Leaves and stems hairy………………..………………..………………..…….....…………..….…..…...… **3**
2. Leaves entire to 5-lobed. Corolla 9.7–12.6(13.9) mm long……….……….……….……..… ***C***. ***hepaticifolia***
  Leaves mostly 5- to 9-lobed. Corolla 8–11 mm long……....….……….……….… ***C***. ***muralis*** subsp. ***muralis***
3. Lower lip of the corolla 8.2–16.6(17.7) mm wide. 9–12 seeds per capsule. Seeds 1.1–1.3 mm long, alveolate to tuberculate……………..……….……….……….……….……….……….……….……....... ***C***. ***muelleri***
  Lower lip of the corolla 4.2–13 mm wide. 4–56 seeds per capsule. Seeds 0.6–1.1(1.2) mm long, smooth to alveolate….....……………..……….……….……….……….……….……….……….……..…….……… **4**
4. Stems, petioles and pedicels highly fragile when fresh, villous. Corolla white to pale violet, ratio spur length/spur width 0.9–2.8. Seeds 0.6–0.9 mm long, smooth to finely alveolate………………...… ***C***. ***fragilis***
  Stems, petioles and pedicels not fragile, sub-glabrous to villous. Corolla pale violet to violet or pink, ratio spur length/spur width 1.6–5.6. Seeds 0.8–1.1 (1.2) mm long, alveolate………...….……… ***C***. ***aequitriloba***

## Methods

### Plant material and DNA extraction

Leaf material for molecular analyses was collected in the field in 2012 and 2013, dried and stored in silica gel. The sampling localities are shown in Fig. 1 and voucher details are provided in Supplementary Table S1. Total genomic DNA was extracted from ca. 10–30 mg silica gel dried leaf material following the CTAB protocol^91^ with some modifications^92^. When less than 10 mg of dried material was available, no sorbitol washing was applied.

### AFLP fingerprinting

AFLP fingerprinting was performed with usually eight to ten individuals per population (Supplementary Table S1), using *C*. *microcalyx* (Boiss.) Wettst. and *C*. *pubescens* (C.Presl) Cufod. as outgroup taxa. AFLP profiles were generated following established protocols^93^ with modifications^94,95^. Three blanks (DNA replaced by water) were included to test for contamination, and 32 samples (10.5%) were used as replicates between PCR batches to test the reproducibility of AFLP fingerprinting. Based on an initial primer trial the following three selective primer combinations were chosen for selective PCR (fluorescent dye in brackets): EcoRI (6-FAM)ACA/MseI-CAT, EcoRI (VIC)AGG/MseI-CAC, and EcoRI (NED)AAC/MseI-CAG (6-FAM labelled primers: Sigma-Aldrich; NED and VIC labelled primers: Applied Biosystems). Selective PCR products were purified using Sephadex G-50 Fine (GE Healthcare Bio-Sciences, Uppsala, Sweden) applied to a MultiScreen-HV plate (Millipore, Molsheim, France) in three steps of 200 μl each and packed at 600 g for 1, 1 and 5 min, respectively. Then 0.8 µl of the elution product was mixed with 10 µl formamide (Applied Biosystems) and 0.125 µl GeneScan 500 ROX (Applied Biosystems) and run on an ABI 3130 automated capillary sequencer at the Department of Botany & Biodiversity Research of the University of Vienna, Austria.

### Analyses of AFLP data

Electropherograms were analysed with Peak Scanner version 1.0 (Applied Biosystems) using default peak detection parameters except employing light peak smoothing. The minimum fluorescent threshold was set to 50 relative fluorescence units. Automated binning and scoring of the AFLP fragments were performed using RawGeno 2.0-1^96^ for R 2.15.0^97^ with the following settings: scoring range 140–500 bp, minimum intensity 50 relative fluorescence units, minimum bin width 1 bp, and maximum bin width 1.5 bp. We chose not to score AFLP fragments shorter than 140 bp as short fragments are more prone to homoplasy than long ones^98^. Fragments with a reproducibility lower than 80% based on sample-replicate comparisons were eliminated. The error rate^39^ was calculated as the ratio of mismatches (scoring 1 versus 0) over phenotypic comparisons in AFLP profiles of replicated individuals. Fragments present/absent in only one individual were excluded.

A Neighbour-Joining (NJ) analysis based on Nei-Li genetic distances^99^ was conducted and bootstrapped (2000 pseudo-replicates) with TREECON v.1.3b^100^.

As an alternative approach to infer the relationships among the studied species, we conducted a species tree analysis using SNAPP^101^. SNAPP is a Bayesian MCMC sampler, which interfaces with the BEAST package^102^ to estimate species trees using a coalescent multispecies approach from biallelic input data. Due to the long run times, we reduced the sampling to two individuals per population. Three MCMCs were initially intended to run for 10 million generations, sampling trees every 1000 generations. However, runs were stopped before reaching the final generation number after checking the convergence of most parameters in Tracer 1.6.0^103^, due to the long run times. Therefore, the first MCMC was run for 153000 generations, the second for 126000 generations, and the third for 392000 generations. After discarding the first 10% of trees of each run, the resulting 980 trees were combined with LogCombiner v.1.8.2, and the topologies were visualized in DensiTree v2.2.5^104^.

A Bayesian phylogeographical diffusion model in continuous space^105^ was performed on the AFLP dataset using BEAST v1.8.2^106^ to infer the main pattern of colonization between the Balearic Islands and Corsica-Sardinia. Geographic input data were coordinates of each population (Supplementary Table S1). We used a simple substitution model with estimated state frequencies for phylogeny inference and the Bayesian skyline coalescent prior^107^ and a piecewise-linear skyline model was set to model population growth. A strict molecular clock was used due to the simple presence-absence structure of AFLP data. The diffusion process was modelled by a lognormal relaxed random walk process, which is an extension of a phylogenetic Brownian motion process that rescales the precision matrix by a branch-specific scalar that is drawn independently from an identical distribution^108^, in our case a lognormal distribution centred on 1. We specified a prior exponential distribution on the standard deviation of the lognormal distribution with a mean of 5. We added random jitter with a window size 1.0 to the tips, as more individuals were sampled from the same location. The analysis of the diffusion inference was run for 200 million generations, logging parameters every 5,000 generations. The performance of the analysis as well as mixing and effective sample size values for all parameters were checked in Tracer 1.6.0^109^. A maximum clade credibility tree (MCC) was produced and annotated with Tree Annotator (part of the BEAST package) after removing 25% of trees as burnin and visualized with FigTree 1.4.2. The diffused MCC tree with annotated diffusion estimates was visualized in SPREAD v.1.0.6^110^ and projected together with polygons representing ancestral areas using ArcGIS 10.3.

Analyses of molecular variance (AMOVAs) and genetic diversity were computed with ARLEQUIN 3.5. Groups were defined according to taxa and – for *C*. *aequitriloba* – to geographic distribution (islands and archipelagos). Average gene diversity over loci (π_n_) was obtained for populations and groups after removing populations with fewer than four sampled individuals. Comparison of gene diversity among groups was performed with Student’s t-tests after testing for normality with Shapiro-Wilk normality tests.

A separate dataset for *C*. *aequitriloba* was prepared and, after independent automated binning and scoring, analysed in the same way. It comprised all populations of the *C*. *aequitriloba* clade (see Results) plus *C*. *fragilis* populations f8 and f9 as outgroup, based on the results of the NJ analysis for the entire dataset. In addition, we performed nonhierarchical K-means clustering^111^, a model-free clustering approach. We used a script of Arrigo *et al*.^112^ in R^97^ to identify genetically homogeneous groups in a dataset pruned to *C*. *aequitriloba*. We performed 100,000 independent runs (i.e., starting from random points) for each assumed value of K (i.e., the number of groups) ranging from 2 to 10.

### Amplification and sequencing of plastid DNA markers

We amplified and sequenced the plastid *ndhF* region and the *rpl32-trnL*^*(UAG)*^ spacer for three individuals per population, with the exceptions of populations a20 and m1 – for which we could only obtain sequences from two individuals –, and the outgroup (*C*. *muralis* G.Gaertn., B.Mey. & Scherb. and *C*. *pubescens*) – with a single individual per population. We used the primers 3′F^113^, +607^114^ and the internal specific primers ndhF CymbF and ndhF CymbR^12^ for the *ndhF* region and rpl32F and trnL^(UAG)^^115^ for the rpl32-trnL spacer. All reactions were carried out in a MasterCycler Gradient thermocycler (Eppendorf).

For both plastid regions, the reaction mix (total volume 25 μL) contained 9 μL of RedTaq ReadyMix PCR Reaction Mix (Sigma-Aldrich), 1.05 μL bovine serum albumin (BSA; 1 mg mL^−1^; Promega), 1.05 μL of each primer (10 μM) and 1.5 μL template DNA. We followed the PCR profiles described in Galbany-Casals *et al*.^116^ for the *ndhF* and Magauer *et al*.^45^ for the *rpl32-trnL*^*(UAG)*^ spacer.

The quality of the PCR products was checked on 1% TBE agarose gels. Subsequently, the amplification products were purified enzymatically using Exonuclease I and Shrimp Alkaline Phosphatase (Fermentas) according to the manufacturer’s instructions. Sanger sequencing was conducted by a commercial sequencing facility (Eurofins MWG Operon, Munich, Germany) using the primers 3′F, + 607, rpl32F and trnL(UAG). Sequences were examined and aligned by hand using Chromas Lite 2.0 (Technelysium Pty Ltd, Tewantin, Australia) and Mega 6.06^117^. GenBank accession numbers are given in Supplementary Table S1.

### Analyses of sequence data

The alignment of the concatenated plastid markers was analysed using statistical parsimony as implemented in TCS 1.21^118^ with the connection limit set to 95%. For this analysis, an indel longer than 1 bp was reduced to a single base pair column allowing this structural mutation to be counted as single base pair mutation only. Two overlapping, but clearly non-homologous indels were detected and considered different. Differences on the indel length (1 bp) caused by different lengths of poly-T regions were not considered due to the high likelihood of homoplastic evolution^119^. Specimens with missing data in polymorphic positions were excluded to avoid ambiguous haplotype assignments. After these modifications, gaps were treated as fifth character state in this analysis.

Maximum parsimony (MP) analyses as well as MP bootstrap analyses of both data sets were performed using PAUP 4.0b10^120^. Indels were coded as binary characters using the simple indel coding method^121^ using SeqState 1.4.1^122^. The most parsimonious trees were searched heuristically with 1000 replicates of random sequence addition, TBR swapping, and MulTrees on. The swapping was performed on a maximum of 1000 trees (nchuck = 1000). All characters were equally weighted and unordered. The data set was bootstrapped using full heuristics, 1000 replicates, TBR branch swapping, MulTrees option off, and random addition sequence with five replicates. *Cymbalaria pubescens* was used as outgroup based on a previous study^12^.

Bayesian Inference (BI) analysis was performed employing MrBayes v.3.2^123^ applying the GTR + G substitution model proposed by the Akaike information criterion implemented in JModelTest 0.1.1^124^. Indels were coded as binary characters using the simple indel coding method^119^. We generated 10,000 trees running MrBayes for 5,000,000 generations and sampling one of every 500 generations. After ensuring that the Markov chain Monte Carlo (MCMC) reached stationarity, we discarded the first 2500 trees as burn-in.

### Morphometric analyses

Twenty-seven characters were scored on the basis of previous studies on the tribe Antirrhineae^20,125^, of which 18 were quantitative and seven were ordinal (Supplementary Table S4). Given that ordination analyses perform better with quantitative characters^126^, qualititative characters such as stem fragility and leave turgescence, which are diagnostic characters in the group, have not been included in these analyses. In *Cymbalaria*, indumentum and seed ornamentation, treated here as ordinal characters, have been considered of main diagnostic importance^21,23,127,128^. Terminology used for indumentum characters follows Payne^129^ and that for seed characters follows Sutton^20^. Seven characters corresponded to vegetative features, 11 to floral features, and seven to fruit and seed features. For the vegetative characters, three measurements per specimen were averaged when possible. As it was not possible to score both floral and fruit or seed characters for all populations, we analysed two datasets. Dataset 1 included 11 floral and seven vegetative characters and dataset 2 included seven fruit and seven vegetative characters. Dataset 1 comprised 129 specimens from 31 populations and dataset 2 comprised 85 specimens from 33 populations (Supplementary Table S1). All specimens used in these analyses were also included in the molecular analyses, with the exception of *C*. *muelleri* and some individuals from population a5 of *C*. *aequitriloba* included in dataset 1, which were added later in order to provide floral measurements. Floral measurements were performed in the field with a caliper. Features of indumentum, calyx, fruits and seeds were examined under a binocular stereoscopic microscope. The other characters were measured on scanned specimens using Image J^130^. Analyses were conducted using a set of R functions contained in MorphoTools ver. 1.01^131^. Pearson and Spearman correlation coefficients were computed to reveal correlation structure among the characters and to ensure that no strong correlations (>|0.95|) were present. After standardization to zero mean and unit variance, principal component analysis (zero-centred PCA based on a covariance matrix) was applied to datasets 1 and 2 to display the overall variation pattern along the first two components. A canonical discriminant analysis (CDA) was performed on dataset 1 to assess the morphological differentiation among the four taxa. Morphological intermediate specimens between *C*. *fragilis* and *C*. *aequitriloba* from populations f1, f3, f4, f5, f8 and f9 were included in *C*. *fragilis* according to the results of the molecular analyses and the PCA.

## Electronic supplementary material

Supplementary information

## Data Availability

DNA sequences have been deposited in GenBank (see Supplementary Table S1). AFLP data matrix, alignments of concatenated plastid sequences and the xml files with details of the BEAST analyses are available at Dryad: 10.5061/dryad.0cr574s.
