# Supplementary material for: Phylogeography of western Mediterranean Cymbalaria (Plantaginaceae) reveals two independent long-distance dispersals and entails new taxonomic circumscriptions
Source: Sci Rep. 2018 Dec 27;8:18079. doi: 10.1038/s41598-018-36412-1 (PMC6308241; doi:10.1038/s41598-018-36412-1)
Supplement: Supplementary file 1 — Supplementary information [file 41598_2018_36412_MOESM1_ESM.doc]

**SUPPLEMENTARY INFORMATION**

**Phylogeography of western Mediterranean *Cymbalaria* (Plantaginaceae) reveals two independent long-distance dispersals and entails new taxonomic circumscriptions**

Pau Carnicero, Peter Schönswetter, Pere Fraga, Núria Garcia-Jacas, Llorenç Sáez, Mercè Galbany-Casals

**Figure S1.** DensiTree of the SNAPP analysis of AFLP data. The complete tree set (thin lines) and consensus trees (thick lines) are shown. The most common topology is marked in blue (61% of trees) and the second-most common topology is marked in red (30%). Green lines show a topology that achieved 9% of the total number of trees.


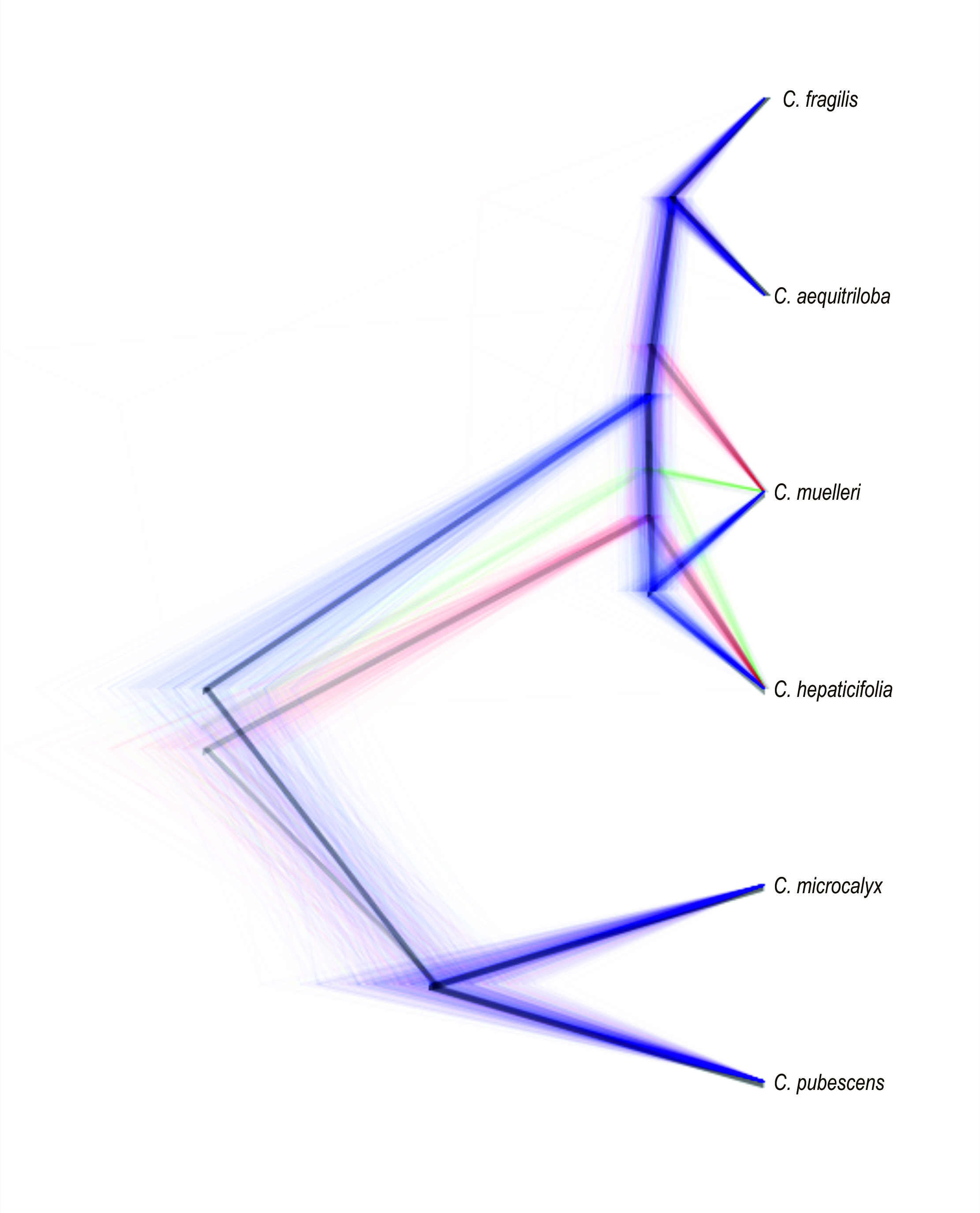


**Figure S2.** Maximum clade credibility (MCC) tree resulting from the Bayesian phylogeographical diffusion model in continuous space performed on the AFLP dataset using BEAST v1.8.2. Populations are numbered as in Fig. 1 and Table S1. Bayesian posterior probabilities ≥ 0.95 are indicated above branches.

**
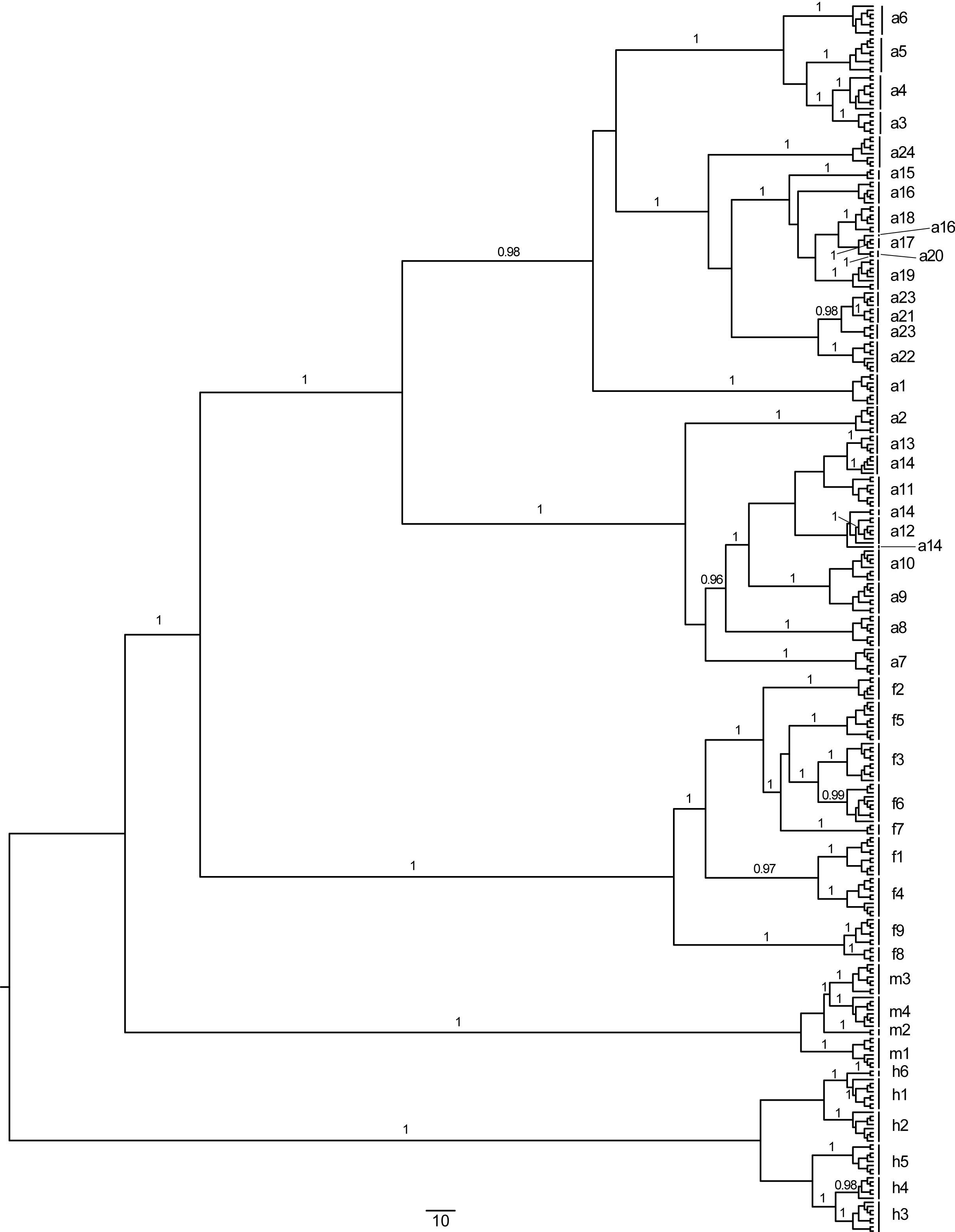
**

**Figure S3.** Ordination diagrams of principal component analysis of 14 reproductive and vegetative morphometric characters obtained for 85 specimens (dataset 2) of *C. aequitriloba* (empty circles), *C. fragilis* (grey circles), *C. hepaticifolia* (black circles) and *C. muelleri* (crosses). Grey circles with thicker outline indicate *C. fragilis* specimens previously considered *C. aequitriloba*. (**a**) Scatter plot of principal component scores for the first two components of morphological variation. (**b**) Relationships of characters projected in the same ordination space as the samples (Lf, leaf; Pd, pedicel; Pt, petiole).

**
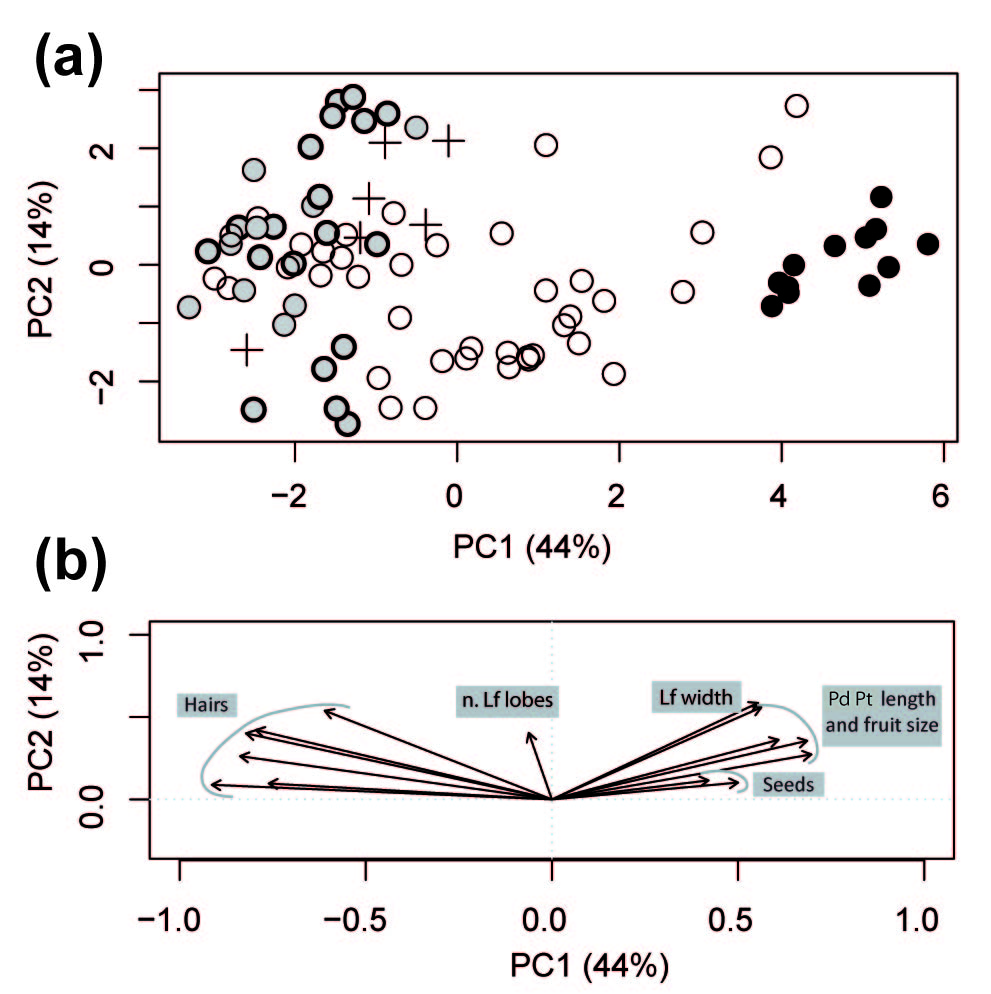
**

**Table S1.** List of sampled populations with information on the population codes used in the text and figures, locality, herbarium voucher, number of individuals included in each analysis and accession numbers. Superscripts next to the haplotype code indicate the number of individuals owing the corresponding haplotype.

| species | population code | voucher | locality | Sampled specimens | | | | Accession numbers | |
| --- | --- | --- | --- | --- | --- | --- | --- | --- | --- |
|  |  |  |  | AFLPs | Haplotypes | Morphology dataset 1 | Morphology dataset 2 | *ndhF* | *rpl32-trnL* |
| *Cymbalaria aequitriloba* | a1 | *P. Carnicero 380 & M. Galbany-Casals* (BC 955777) | IT, Sardinia, Badde Salighes, Ortachis | 8 | H22^3^ | 7 | 1 | MK217524 - MK217526 | MK217654 - MK217656 |
| *Cymbalaria aequitriloba* | a2 | *P. Carnicero 385 & M. Galbany-Casals* (BC 955774) | IT, Sardinia, Badde Urbara, Mt Pertusu | 7 | H8^3^ | 5 | 1 | MK217527 - MK217529 | MK217657 - MK217659 |
| *Cymbalaria aequitriloba* | a3 | *P. Carnicero 396 & M. Galbany-Casals* (BC 955775) | IT, Sardinia, Seui, Funtana dorada | 6 | H19^3^ | 0 | 1 | MK217530 - MK217532 | MK217660 - MK217662 |
| *Cymbalaria aequitriloba* | a4 | *P. Carnicero 412 & M. Galbany-Casals* (BC 955776) | IT, Sardinia, Seui, Perda Liana | 9 | H26^3^ | 1 | 3 | MK217533 -MK217535 | MK217663 - MK217665 |
| *Cymbalaria aequitriloba* | a5 | *P. Carnicero 415 & M. Galbany-Casals* (BC 955781) | IT, Sardinia, Gadoni, Foresta comunale | 9 | H22^3^ | 0 | 4 | MK217536 - MK217538 | MK217666 - MK217668 |
|  | a5 | *P. Carnicero 1232 & M. Unzeta* (BC 955767) |  |  |  | 2 | 0 |  |  |
| *Cymbalaria aequitriloba* | a6 | *P. Carnicero 421 & M. Galbany-Casals* (BC 955768) | IT, Sardinia, Bruncu Spina, ski resort | 8 | H26^2^ | 4 | 1 | MK217539 - MK217541 | MK217669 - MK217671 |
| *Cymbalaria aequitriloba* | a7 | *P. Carnicero 424 & M. Galbany-Casals* (BC 955768) | IT, Sardinia, Oliena, Sorgenti del Gologone | 7 | H13^3^ | 1 | 3 | MK217542 - MK217544 | MK217672 - MK217674 |
| *Cymbalaria aequitriloba* | a8 | *P. Carnicero 425 & M. Galbany-Casals* (BC 955779) | IT, Sardinia, Témpio Pausania, Mt Limbara | 8 | H5^1^, H8^2^ | 2 | 3 | MK217545 - MK217547 | MK217675 - MK217677 |
| *Cymbalaria aequitriloba* | a9 | *P. Carnicero 430 & M. Galbany-Casals* (BC 955780) | FR, Corsica, Bastia, summit of Pigno | 8 | H6^1^, H8^2^ | 7 | 5 | MK217548 - MK217550 | MK217678 - MK217680 |
| *Cymbalaria aequitriloba* | a10 | *P. Carnicero 432 & M. Galbany-Casals* (BC 955778) | FR, Corsica, Sisco, W of Mte Corvu | 8 | H7^3^ | 0 | 1 | MK217551 - MK217553 | MK217681 - MK217683 |
| *Cymbalaria aequitriloba* | a11 | *P. Carnicero 446 & M. Galbany-Casals* (BC 955773) | FR, Corsica, 3km above Albertacce, left edge of Golo | 8 | H8^2^, H10^1^ | 1 | 1 | MK217554 - MK217556 | MK217684 - MK217686 |
| *Cymbalaria aequitriloba* | a12 | *P. Carnicero 448 & M. Galbany-Casals* (BC 955772) | FR, Corsica, Paisolu Aitone, Foret Demaniale d'Aitone | 7 | H9^3^ | 2 | 1 | MK217557 -MK217559 | MK217687 - MK217689 |
| *Cymbalaria aequitriloba* | a13 | *P. Carnicero 451 & M. Galbany-Casals* (BC 955771) | FR, Corsica, from Zicavu to Guitera les Bains | 5 | H9^2^ | 0 | 1 | MK217560 - MK217562 | MK217690 - MK217692 |
| *Cymbalaria aequitriloba* | a14 | *P. Carnicero 460 & M. Galbany-Casals* (BC 955770) | FR, Corsica, Bocca Illarata | 8 | H6^3^ | 0 | 0 | MK217563 - MK217565 | MK217693 - MK217695 |
| *Cymbalaria aequitriloba* | a15 | *P. Carnicero 544 et al.* (BC 955766) | SP, Balearic Islands, Menorca, Ciutadella, Cala en Turqueta | 3 | H4^3^ | 0 | 0 | MK217566 - MK217568 | MK217696 - MK217698 |
| *Cymbalaria aequitriloba* | a16 | *P. Carnicero 562 et al.* (BC 955762) | SP, Balearic Islands, Menorca, Ferreries, Ermita | 7 | H4^3^ | 1 | 0 | MK217569 - MK217571 | MK217699 - MK217701 |
| *Cymbalaria aequitriloba* | a17 | *P. Carnicero 563 et al.* (BC 955761) | SP, Balearic Islands, Menorca, Es Mercadal, Llinàritx Nou | 3 | H4^3^ | 0 | 0 | MK217572 - MK217574 | MK217702 - MK217704 |
| *Cymbalaria aequitriloba* | a18 | *P. Carnicero 571 et al.* (BC 955764) | SP, Balearic Islands, Menorca, Es Mercadal, Toro | 7 | H2^3^ | 1 | 1 | MK217575 - MK217577 | MK217705 - MK217707 |
| *Cymbalaria aequitriloba* | a19 | *P. Carnicero 574 et al.* (BC 955763) | SP, Balearic Islands, Menorca, Binifabini | 8 | H4^2^ | 0 | 0 | MK217578 - MK217580 | MK217708 - MK217710 |
| *Cymbalaria aequitriloba* | a20 | *P. Carnicero 579 et al.* (BC 955765) | SP, Balearic Islands, Menorca, Es Grau | 2 | H4^2^ | 1 | 0 | MK217581, MK217582 | MK217711, MK217712 |
| *Cymbalaria aequitriloba* | a21 | *L. Sáez 7365* (BC 955759) | SP, Balearic Islands, Mallorca, Sóller, Gorg Blau | 4 | H4^3^ | 3 | 2 | MK217589 - MK217591 | MK217718 - MK217720 |
| *Cymbalaria aequitriloba* | a22 | *L. Sáez 7366 & X. Rotllan* (BC 879621) | SP, Balearic Islands, Mallorca, Formentor | 8 | H4^3^ | 0 | 4 | MK217583 - MK217585 | KP851095, MK217713, MK217714 |
| *Cymbalaria aequitriloba* | a23 | *L. Sáez 7368* (BC 955758) | SP, Balearic Islands, Mallorca, Sóller, Puig Major | 8 | H3^3^ | 5 | 4 | MK217592 - MK217594 | MK217721 - MK217723 |
| *Cymbalaria aequitriloba* | a24 | *L. Sáez 7385* (BC 955760) | SP, Balearic Islands, Mallorca, Calvià, Puig de Galatzó, vessant NE | 8 | H4^3^ | 10 | 0 | MK217586 - MK217588 | MK217715 - MK217717 |
| *Cymbalaria fragilis* | f1 | *P. Carnicero 353 & M. Galbany-Casals* (BC 955790) | SP, Balearic Islands, Menorca, Fornells, Mola de Fornells | 10 | H4^3^ | 5 | 0 | MK217595 - MK217597 | MK217724 - MK217726 |
| *Cymbalaria fragilis* | f2 | *P. Carnicero 800 & P. Fraga* (BC 955791) | SP, Balearic Islands, Menorca, Ferreries, Barranc d Algendar, Pas d en Revull | 6 | H4^3^ | 4 | 2 | MK217598 - MK217600 | MK217727 - MK217729 |
| *Cymbalaria fragilis* | f3 | *P. Carnicero 805 & P. Fraga* (BC 955792) | SP, Balearic Islands, Menorca, Es Migjorn, Barranc Albranca | 10 | H4^3^ | 5 | 0 | MK217601 - MK217603 | MK217730 - MK217732 |
| *Cymbalaria fragilis* | f4 | *P. Carnicero 810 & P. Fraga* (BC 955793) | SP, Balearic Islands, Menorca, Maó, Mola de Maó | 10 | H11^3^ | 5 | 4 | MK217605 - MK217607 | MK217733 - MK217735 |
| *Cymbalaria fragilis* | f5 | *P. Carnicero 814 & P. Fraga* (BC 955794) | SP, Balearic Islands, Menorca, Barranc de Binigaus, St. Agustí Vell | 10 | H4^3^ | 5 | 5 | MK217608 -MK217610 | MK217736 - MK217738 |
| *Cymbalaria fragilis* | f6 | *P. Carnicero 816 & P. Fraga* (BC 955795) | SP, Balearic Islands, Menorca, Barranc de Trebalúger, Son Marcer de Baix | 10 | H4^3^ | 5 | 4 | MK217611 - MK217613 | MK217739 - MK217741 |
| *Cymbalaria fragilis* | f7 | *P. Carnicero 818 & P. Fraga* (BC 955796) | SP, Balearic Islands, Menorca, between Binimassó and Algendaret, Canal d'en Curt | 3 | H1^3^ | 4 | 3 | MK217614 - MK217616 | MK217742 - MK217744 |
| *Cymbalaria fragilis* | f8 | *L. Sáez 7329* (BC 955797) | SP, Balearic Islands, Cabrera, Imperialet | 4 | H4^3^ | 0 | 1 | MK217620 - MK217622 | MK217748 - MK217750 |
| *Cymbalaria fragilis* | f9 | *L. Sáez 7377* (BC 879620) | SP, Balearic Islands, Cabrera, Es Llenegall | 7 | H4^3^ | 2 | 4 | MK217617 - MK217619 | MK217745 - MK217747 |
| *Cymbalaria hepaticifolia* | h1 | *P. Carnicero 427 & M. Galbany-Casals* (BC 955753) | FR, Corsica, NE of Col de Bavella | 8 | H9^3^ | 0 | 0 | MK217623 - MK217625 | MK217751 - MK217753 |
| *Cymbalaria hepaticifolia* | h2 | *P. Carnicero 433 & M. Galbany-Casals* (BC 955757) | FR, Corsica, Sisco, Mt Corvu | 8 | H16^3^ | 4 | 3 | MK217626 - MK217628 | MK217754 - MK217756 |
| *Cymbalaria hepaticifolia* | h3 | *P. Carnicero 441 & M. Galbany-Casals* (BC 955754) | FR, Corsica, Castagniccia, Felce | 8 | H15^2^ | 0 | 1 | MK217629 - MK217631 | MK217757 - MK217759 |
| *Cymbalaria hepaticifolia* | h4 | *P. Carnicero 444 & M. Galbany-Casals* (BC 879631) | FR, Corsica, Castagniccia, road from Croce to Porta | 7 | H15^3^ | 6 | 3 | MK217632 - MK217634 | MK217760 - MK217762 |
| *Cymbalaria hepaticifolia* | h5 | *P. Carnicero 447 & M. Galbany-Casals* (BC 955755) | FR, Corsica, Albertacce, Foresta de Valdo niello, close to sorgenti di mezzanule | 8 | H12^4^, H14^1^, H15^1^ | 7 | 5 | MK217635 - MK217637 | MK217763 - MK217765 |
| *Cymbalaria hepaticifolia* | h6 | *P. Carnicero 456 & M. Galbany-Casals* (BC 955756) | FR, Corsica, N of Bocca d'Illarata | 2 | H17^2^ | 0 | 1 | MK217638 - MK217640 | MK217766 - MK217768 |
| *Cymbalaria muelleri* | m1 | *P. Carnicero 389 & M. Galbany-Casals* (BC 879630) | IT, Sardinia, Ulassai, Bruncu Matzeu | 8 | H22^1^ | 0 | 3 | MK217641, MK217642 | MK217769, MK217770 |
|  | m1 | *P. Carnicero 1263 & M. Unzeta* (BC 955784) |  |  |  | 6 | 0 |  |  |
| *Cymbalaria muelleri* | m2 | *P. Carnicero 406 & M. Galbany-Casals* (BC 879629) | IT, Sardinia, Seui, Foresta de Montarbu, SE cliffs of Genni d'Acca | 2 | H24^1^, H25^1^ | 0 | 0 | MK217643 - MK217645 | MK217771 - MK217773 |
|  | m2 | *P. Carnicero 1251 & M. Unzeta* (BC 955783) |  |  |  | 6 | 0 |  |  |
| *Cymbalaria muelleri* | m3 | *P. Carnicero 414 & M. Galbany-Casals* (BC 955485) | IT, Sardinia, Laconi, Corona Sa Guardia, S'aza du Ziu Chiccu | 8 | H18^3^ | 0 | 2 | MK217646 - MK217648 | MK217774 - MK217776 |
|  | m3 | *P. Carnicero 1223 & M. Unzeta* (BC 955789) |  |  |  | 6 | 0 |  |  |
| *Cymbalaria muelleri* | m4 | *P. Carnicero 416 & M. Galbany-Casals* (BC 955786) | IT, Sardinia, Gadoni, Foresta comunale | 8 | H20^1^, H21^1^, H23^1^ | 0 | 1 | MK217649 - MK217651 | MK217777 - MK217779 |
|  | m4 | *P. Carnicero 1231 & M. Unzeta* (BC 955788) |  |  |  | 6 | 0 |  |  |
| outgroups | | | | | | | | | |
| *Cymbalaria microcalyx* |  | *P. Carnicero 1074* (BC 955798) | GR, Peloponnese, Lakonia, road from Trypi to Kalamata | 1 |  |  |  |  |  |
| *Cymbalaria muralis* |  | *P. Carnicero 420 & M. Galbany-Casals* (BC 955752) | IT, Sardinia, Aritzo |  |  |  |  | MK217652 | MK217780 |
| *Cymbalaria pubescens* |  | *M. Galbany-Casals 2311 & N. Garcia-Jacas* (BC 955799) | IT, Sicily, from Portella della Páglia to La Pizzuta | 2 |  |  |  | MK217653 | MK217781 |

**Table S2.** Hierarchical AMOVAs. BI: Balearic Islands, Co: Corsica, Sa: Sardinia.

|  | **Groups** | **Among groups** | | **Among populations** | | | **Within populations** | |
| --- | --- | --- | --- | --- | --- | --- | --- | --- |
|  |  | **d.f.** | **% variation** | **d.f.** | **% variation** | **d.f.** | | **% variation** |
| Western Mediterranean *Cymbalaria* | 4 (species) | 3 | 28.77 | 39 | 36.47 | 257 | | 34.76 |
| *C. aequitriloba* | 2 (BI, Co-Sa) | 1 | 24.71 | 21 | 33.55 | 133 | | 41.74 |
|  | 3 (BI, Co, Sa) | 2 | 28.08 | 20 | 27.88 | 133 | | 44.05 |
| *C. aequitriloba* (BI) | - | - | - | 9 | 35.53 | 48 | | 64.47 |
| *C. aequitriloba* (Co) | - | - | - | 5 | 27.65 | 38 | | 72.35 |
| *C. aequitriloba* (Sa) | - | - | - | 7 | 44.66 | 54 | | 55.34 |

**Table S3.** Average gene diversity (π_n_ ) and Student's t-tests for comparisons of gene diversity among groups. BI: Balearic Islands, Co: Corsica, Sa: Sardinia.

| **Taxon (group of populations)** | **Average gene diversity (π_n_ )** | **t-test** | | |  |
| --- | --- | --- | --- | --- | --- |
|  |  | **vs.** | **T** | **p-value** |  |
| *C. aequitriloba* | 0.090 |  |  |  |  |
| *C. aequitriloba* (BI) | 0.067 | *C. aequitriloba* (Sa) | -5.536 | 0.000 * |  |
|  |  | *C. aequitriloba* (Co) | -3.528 | 0.005 * |  |
| *C. aequitriloba* (Co) | 0.101 | *C. aequitriloba* (Sa) | -0.566 | 0.58 |  |
| *C. aequitriloba* (Sa) | 0.107 |  |  |  |  |
| *C. fragilis* | 0.079 | *C. aequitriloba* | 1.439 | 0.17 |  |
|  |  | *C. aequitriloba* (BI) | -1.532 | 0.15 |  |
| *C. hepaticifolia* | 0.104 | *C. aequitriloba* | -0.540 | 0.62 |  |
|  | 0.104 | *C. aequitriloba* (Co) | -0.119 | 0.91 |  |
| *C. muelleri* | 0.092 | *C. aequitriloba* | -0.244 | 0.82 |  |
|  |  | *C. aequitriloba* (Sa) | 1.6 | 0.19 |  |

**Table S4.** Characters studied in morphometric analyses.

| **Character** | **Type** | **dataset** |
| --- | --- | --- |
| Upper lip width (mm) | Quantitative | 1 |
| Upper lip length (mm) | Quantitative | 1 |
| Upper lip sinus (mm) | Quantitative | 1 |
| Lower lip width (mm) | Quantitative | 1 |
| Lower lip length (mm) | Quantitative | 1 |
| Corolla length (mm) | Quantitative | 1 |
| Corolla tube width (mm) | Quantitative | 1 |
| Spur length (mm) | Quantitative | 1 |
| Spur width (mm) | Quantitative | 1 |
| Indumentum of capsule | Ordinal (0 = glabrous; 1 = subglabrous, sparsely distributed hairs; 2 = non-overlapping uniformly distributed hairs; 3 = overlapping hairs) | 2 |
| Indumentum of stem |  | 1, 2 |
| Indumentum of upper leaf surface |  | 1, 2 |
| Indumentum of lower leaf surface |  | 1, 2 |
| Indumentum of calyx |  | 1, 2 |
| Indumentum of upper part of pedicel |  | 1, 2 |
| Number of leaf lobes | Quantitative | 1, 2 |
| Maximum leaf width (mm) | Quantitative | 1, 2 |
| Pedicel length at anthesis (mm) | Quantitative | 1 |
| Pedicel length, fruiting (mm) | Quantitative | 2 |
| Petiole length of bracts at anthesis (mm) | Quantitative | 1 |
| Petiole length of bracts, fruiting (mm) | Quantitative | 2 |
| Calyx length (mm) | Quantitative | 2 |
| Capsule length (mm) | Quantitative | 2 |
| Seed surface | Ordinal (1 = smooth; 2 = alveolate; 3 = cristate-alveolate; 4 = cristate; 5= tuberculate) | 2 |
| Seed length (mm) | Quantitative | 2 |
